# Supplementary material for: CRISPR/Cas9-Mediated Models of Retinitis Pigmentosa Reveal Differential Proliferative Response of Müller Cells between Xenopus laevis and Xenopus tropicalis
Source: Cells. 2022 Feb 25;11(5):807. doi: 10.3390/cells11050807 (PMC8909648; doi:10.3390/cells11050807)
Supplement: Supplementary file 1 [file cells-11-00807-s001.zip › cells-1539079-supplementary.pdf]

A

B

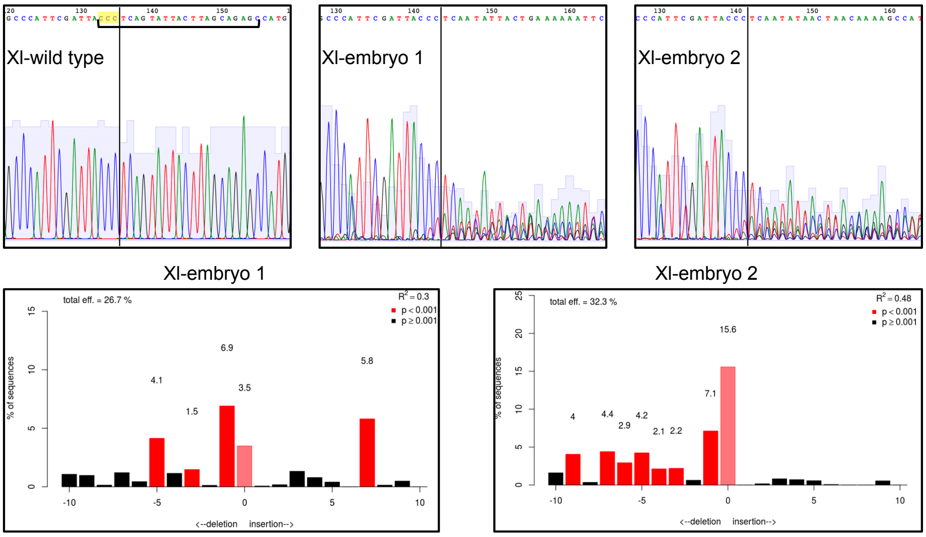

C

	Nucleotidic sequence	InDels	Protein sequence	Size
wt-*rho.L*	TACGAAGCCCATTCGATTA**CCC**TCAGTATTACTTAGCAGAGCCATG		...VVRSPFDYPQYYLAEPWQYSALAA...	354
wt-*rho.S*	TGCGAAGCCCCTTTGATTA**CCC**TCAGTATTACTTAGCAGAGCCATG			
wt-rho.2.L	TACGAAGCCCATTCGATTA**CCC**TCAGTATTACTTAGCAGAGCCATG			
Mix1-seq1	TACGAAGCCCATTCGATTA**CCC**----TATTACTTAGCAGAGCCATGG	-4	...VVRSPFDYPIT*	29
Mix1-seq2	TACGAAGCCC----GATTA**CCC**TCA--------TAGCAGAGCCATGG	-12	...VVRSP--ITLI--AEPWQYSALAA...	350
Mix1-seq3	TACGAAGCCCATTCGATTA**C**------------TTAGCAGAGCCATGG	-12	...VVRSPFDY----LAEPWQYSALAA...	350
Embryo1-seq1	TACGAAGCCCATTCGATTA**CCC**TCA-T-TTACTTAGCAGAGCCATG	-2	...VVRSPFDYPHLLSRAMAIF...WLLH*	111
Embryo1-seq2	TACGAAGCCCATTCGATTA**CCC**-CA---TTACTTAGCAGAGCCATG	-4	...VVRSPFDYPIT*	29
Embryo1-seq3	TGCGAAGCCCCTTTGATTA**CCC**TCAG----------------------<br>---------CACT	-31	...VVRSPFDYPQHWLLTCSCSSCLGYQSTS*	46
Embryo1-seq4	TGCGAAGCCCCTTTGATTA**CCC**TTTTATTACTTAGCAGAGCCATG	-3;+3	...VVRSPFDYPFYYLAEPWQYSALAAY...	354
Embryo2-seq1	TACGAAGCCCATTCGATTA**CCC**T----ATTACTTAGCAGAGCCATG	-4	...VVRSPFDYPIT*	29
Embryo2-seq2	TACGAAGCCCATTCGATTA**CCC**T----ATTACTTAGCAGAGCCATG	-4	...VVRSPFDYPIT*	29
Embryo2-seq3	TACGAAGCCCATTCGATTA**CCC**TCAGTATTACTTAGCAGAGCCATG	wt	...VVRSPFDYPQYYLAEPWQYSALAA...	354
Embryo2-seq4	TACGAAGCCCATTCGATTA**CCC**T------TACTTAGCAGAGCCATG	-6	...VVRSPFDYP--YLAEPWQYSALAA...VSPA*	352
Embryo3-seq1	TGCGAAGCCCCTTTGATTA**CCC**T-GGTATTACTTAGCAGAGCCATG	-2;+1	...VVRSPFDYPGIT*	30
Embryo3-seq2	TGCGAAGCCCCTTTGATTA**CCC**T----ATTACTTAGCAGAGCCATG	-4	...VVRSPFDYPIT*	29
Embryo3-seq3	TACGAAGCCCATTCGATTA**C**------------TTAGCAGAGCCATG	-12	...VVRSPFDY----LAEPWQYSALAA...VSPA*	350
Embryo3-seq4	TACGAAGCCCATTCGATTA**CCC**----------TTAGCAGAGCCATG	-10	...VVRSPFDYP*	27
Embryo3-seq5	TGCGAAGCCCCTTTGATTA**CCC**TCAG-------------AGCCATG	-13	...VVRSPFDYPQSHGNIQHWLL...GYQSTS*	52

Figure S1. Genomic analysis of *X. laevis rho* crispants. (A) Sequencing chromatograms showing the *rho* sequences of one *X. laevis* wild type embryo and two *rho* crispants. The bracket delineates the *rho* sgRNA target region. The PAM is in yellow. **(B)** TIDE analysis of the sequences presented in A. Note the presence of multiple Indels in these crispant individuals. **(C)** Sequences obtained from individual clones of PCR products amplified from genomic DNA. One mixture of *Xenopus laevis* F0 embryos (Mix1) or 3 single embryos were used. For each clone, the targeted sequence is shown in pink with PAM in bold, while indels are in blue. The right column indicates the corresponding protein sequence and its size (number of amino acids).

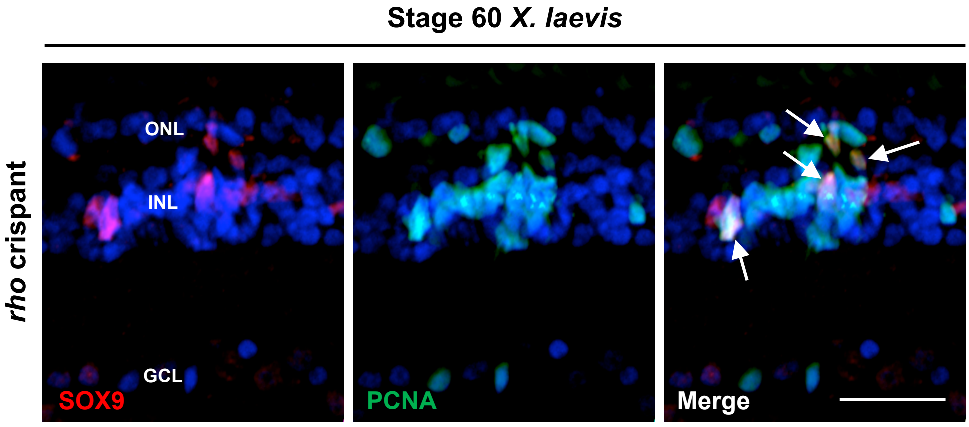

Figure S2. Analysis of proliferative cells in *X. laevis rho* crispants. Typical sections from stage 60 crispant *X. laevis* tadpoles, immunostained for PCNA (a marker of proliferative cells) and SOX9 (a marker of Müller cells). Arrows point to double positive cells. Cell nuclei are counterstained with Hoechst. GCL: Ganglion Cell Layer, INL: Inner Nuclear Layer, ONL: Outer Nuclear Layer. Scale bar: 25 µm.

Supplementary Table S1. Primers

Primers for genotyping in *X. tropicalis*	Primers for genotyping in *X. laevis*
F: TGGCAGCTTACTTCAGGTGG	F: TGGGATCACAGGCTTCTAGG
R: TGTAGCAACCAGTTTGGCCA	R: TGTAGCAACCAGTTTGGCCA

Supplementary Table S2. List of antibodies

	Species	Reference	Dilution
**Primary Antibody**			
Anti- Rhodopsin	Mouse	MABN15, Millipore	1:1000
Anti- S-opsin	Rabbit	AB5407, Millipore	1:500
Anti- M-opsin	Rabbit	AB5405, Millipore	1:500
Anti- Calbindin	Rabbit	PC253L, Calbiochem	1:100
Anti- BrdU	Rat	Ab6326, Abcam	1:400
Anti- Recoverin	Rabbit	AB5585, Millipore	1:1000
Anti- Sox9	Rabbit	AB5535, Millipore	1:100
Anti-cleaved Caspase3	Rabbit	9661S, Cell signaling	1:300
Anti -YAP	Rabbit	Ab3961, Abcam	1:50
Anti PCNA	Mouse	M0879, Dako	1:500
**Secondary Antibody**			
Alexa Fluor 594 anti-mouse		A11005, Thermo Fisher Scientific	1:1000
Alexa Fluor 488 anti-mouse		A11001, Thermo Fisher Scientific	1:1000
Alexa Fluor 488 anti-mouse highly cross-adsorbed (used in combination with anti-rat)		A11029, Thermo Fisher Scientific	1:1000
Alexa Fluor 594 anti-rabbit		A11012, Thermo Fisher Scientific	1:1000
Alexa Fluor 488 anti-rabbit		A11008, Thermo Fisher Scientific	1:1000
Alexa Fluor 594 anti-rat		A11007, Thermo Fisher Scientific	1:1000
